# A Movie Recommender System Based on User Profile and Artificial Bee Colony Optimization

**DOI:** 10.1155/2023/2311817

**Published:** 2023-10-25

**Authors:** Faezeh Rajabi Kouchi, Sahar Oftadeh Balani, Amirhossein Esmaeilpour, Masoume Shafieian, Rzgar Sirwan, Adil Hussein Mohammed

**Affiliations:** ^1^Department of Computer Engineering, Central Tehran Branch, Islamic Azad University, Tehran, Iran; ^2^Department of Computer Science, Yadegar-e-Imam Khomeini (RAH) Shahre Rey Branch, Islamic Azad University, Tehran, Iran; ^3^Computer Engineering Department, Shomal University, Amol, Iran; ^4^IRIBU University, Department of Technology and Media Engineering, Tehran, Iran; ^5^Department of Computer Science, College of Science and Technology, University of Human Development, Sulaymaniyah, Iraq; ^6^Department of Communication and Computer Engineering, Faculty of Engineering, Cihan University-Erbil, Kurdistan Region, Erbil, Iraq

## Abstract

In this study, a new algorithm for recommending movies to viewers has been proposed. To do this, the suggested method employs data mining techniques. The proposed method includes three steps for generating recommendations: “preprocessing of user profile information,” “feature extraction,” and “recommendation.” In the first step of proposed method, the user information will be examined and transformed into a form that can be handled in the next phases. In the second step of the proposed method, user attributes are then extracted as a collection of their individual qualities, as well as the average rating of each user for various genres. The bee colony optimization algorithm is then used to select the optimal features. Finally, in the third step of the proposed method, the ratings of similar users are utilized to offer movies to the target user, and the similarities between various users are determined using the characteristics calculated for them, as well as the Euclidean distance criteria. The proposed method was evaluated using the MovieLens database, and its output was assessed in terms of precision and recall criteria; these results show that the proposed method will increase the precision by an average of 1.39% and the recall by 0.8% compared to the compared algorithms.

## 1. Introduction

The Internet is the first and most accessible source of information for users to search for content and obtain information. Today, many websites welcome millions of users with different interests and characteristics on a daily basis. One of the main concerns of the owners of these websites is to provide the appropriate content that the users want in the fastest possible time. This need has led to the formation of recommender systems that can offer the user the desired content, item, or product [1]. Today, recommender systems are one of the most widely used aspects of web analytics and review and analyze user behavioral information in interaction with website in order to predict the future behavior of users and provide an environment tailored to the user's tastes and needs [2]. Since one of the issues in e-commerce is to provide a user-friendly environment, this branch of data mining science has been considered by many researchers and several recommending systems have been proposed for use on the web [3]. Due to the significant increase of information available on the web, users are faced with a large amount of information in their daily interactions. In order to prevent confusion for users and avoid wasting their time when searching for the information they need, recommender systems have been created and facilitate users' access to the required information by personalizing the website environment. To create web recommender systems, the required information is collected through user behavior. This information represents requests made by the user to access specific content on the server. Behavioral information collected from other users is compared with the behavior of a new user who is currently using the website, and a list of suggestions is prepared for the current user. This method of inference is one of the most widely used methods of information filtering in recommender systems [4, 5]. However, in order to achieve an efficient recommender system that meets today's needs, we face two basic problems: first, the modeling of user behavior must be done in an efficient manner and in accordance with computational patterns. The second issue is to prevent the use of redundant and irrelevant information in providing recommendations. In this study, a new method is presented to solve the mentioned problems. The contribution of this study can be summarized as follows:

- The proposed method uses a combination of behavioral characteristics and user profile information to form a user model and provides a computational method for making recommendations based on user-created models. The computational cost of this method is very low and this makes the proposed method a more efficient way to be used in huge networks compared to the previous solutions (the computational cost of proposed method is discussed in Section 4).
- In order to prevent the use of redundant and irrelevant information in recommendation, artificial bee colony (ABC) optimization algorithm is used. The feature selection step can be effective in reducing the processing time (due to reducing the number of features) and improving the precision of the recommender system (due to providing recommendations based on optimal features).

Since the above two cases have not been addressed in previous works, they can be considered as innovations of the proposed method. The remainder of this study is organized as follows: in the second section, the previous works are reviewed and in the third section, the details of the proposed method are presented. In the fourth section, the results of the implementation and evaluation of the proposed method are presented and in the fifth section, conclusions are made. Finally, some suggestions for further research in this regard are provided.

## 2. Related Works

Since recommending systems have been one of the most popular areas of research in recent years, the number of studies conducted in this area is significant. In this section, we will review some of this research. Sun et al. [6] have developed a user-centric system for recommending content to social network users. In this method, the communication between users and their individual behavior in the social network is considered in order to recommend content simultaneously. In this method, groups are formed based on the common interests of users. Then, a collection of user feedback on the content received by users in the same group is collected and similar content is recommended to the user according to his wishes. Yadav et al. [7] presented a film recommendation system using principal component analysis (PCA) and K-means clustering. In this method, principal component analysis is used to preprocess and reduce the dimensions of the features. In this way, the user descriptive features will be created in a compressed capacity. The K-means clustering algorithm is then used to determine the category of users similar to the target user and provide recommendations based on it.

Arojo et al. [8] tried to model the behavior of social media users using methods based on dynamic recommender structure in order to personalize social networks. In this research, preprocessing is first performed on the social network server data in order to prepare the required data for the next step and delete the useless data. Then, two types of classification are performed on the data of the previous step. The first classification tries to identify users based on the pages visited. The second classification tries to determine which user is more interested in which part of the social networks of the site, in fact, in this part, the system intends to identify the interests of users.

The authors in reference [9] believe that preprocessing plays an important role in the dynamic recommender structure. In this study, a similarity criterion to the clustering algorithm is added, which shows that the simulation results show that the similarity of the results is much better than the previous clustering criteria. Dakotor [10] introduced a new similarity criterion for use in recommender systems. The accuracy of motion patterns depends on the similarity criterion considered for the clustering algorithm. Also in this research, two criteria of frequency and duration of user presence on a page have been used. Gorji et al. [11] have proposed a hybrid approach based on application-based dynamic recommender structure and content-based dynamic recommender structure. This system consists of five units. Like many recommendation systems, work begins with preprocessing and is followed by session recognition. Sessions are made by taking the weight of the plates. After the motion patterns are created, these patterns are also combined with the page content using dynamic application-based dynamic recommender structure methods and user profiles. Then, some sort of classification will be done and each of the profiles will be placed in a class, so that suggestions can be generated for users. Gunawardana and Shani [12] have proposed a hybrid recommending system using structure and application data. This method is based on distributed learning automata and also uses page ranking algorithms and graph segmentation. How to work in this method is that first using the application data and the structure of a distributed learning automata determines the similarity between the pages and then using the score ranking algorithm of these pages. It then suggests new pages to users using the Markov model.

Kung et al. [13] have proposed a hybrid recommender system for social networks that can use user and content data and structure data to generate user motion models and use these models to generate suggestions for the site's social network users. Latahabai et al. [14] proposed a system that uses automated rules and indexing to create automated profiles for users using application-based dynamic recommender structure methods. In fact, in this method, profiles are created based on the preprocessing of application data. Parish et al. [15] proposed a method based on a dynamic content-based recommender structure that features a number of repetitions of words and a profile that is automatically created based on user behavior. In this method, the authors weigh the words on the social networks and then, based on an algorithm, remove the words with less weight than the threshold, and thus show each page as a weighted word vector. Then, this concept can be expanded and in fact, each user profile can be displayed in the form of keywords and their weight. Toth and Lengyel [16] tried to improve the user's search results by providing a method without the user explicitly stating them. In fact, the function of this system is such that the user submits his request to the search engine. The recommender system reviews the user's profile and updates it each time so that it can provide more detailed suggestions in subsequent searches. Several nongradient (evolutionary) search strategies have been evaluated by Fozooni et al. [17]. For the purpose of assessing the general efficacy of genetic algorithms, particle swarm optimization, and differential evolution for optimization of mathematical equations, they developed and tested genetic algorithms, particle swarm optimization, and differential evolution.

Optimization algorithms may be used for solving NP-hard problem in different fields. For example, Han et al. [18] tried to minimize the age of information (AoI) in unnamed aircraft vehicle (UAV)-aided vehicular edge computing networks using game theory. Wang et al. [19] proposed a game-based model to optimize the channel access-based AoI minimization problem in UAV-based mobile edge computing networks. Yang et al. [20] proposed a game-based model for AoI optimization in the UAV-aided traffic monitoring network under attack. Akram et al. [21] have developed a deep learning-based technique for Urdu short text clustering. Their deep neural network model learns clustering objectives by converting the high dimensional feature space to a low dimensional feature space. Abbasi et al. [22] have proposed an authorship identification system using ensemble learning. This system uses a count vectorizer and bi-gram term frequency-inverse document frequency (TF-IDF) features to describe the characteristics of the authors. The related works are summarized in Table 1.

## 3. Proposed Method

In this section, we will describe the proposed algorithm for recommending movies to the users. The proposed solution for providing recommendation to users includes three main steps:

1. Preprocessing user profile information
2. Extracting features and constructing user models
3. Providing recommendations based on user models

**Figure 1:**
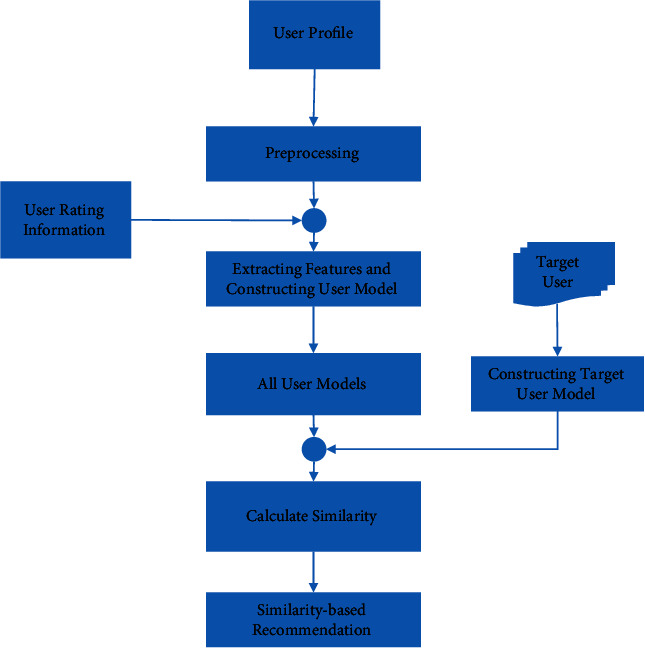
Diagram of the proposed method.

The diagram of the proposed algorithm is shown in Figure 1.

The assumed network database, as illustrated in Figure 1, contains a user profile table and a user rating table for various movie titles, which are used as inputs of the proposed method. Preprocessing the information of the user profiles is the first step of the proposed method. The personal information of each user will be translated into a format that can be handled by the proposed technique throughout this procedure. The proposed technique then uses a model structure to define the attributes of the retrieved user profile information as well as the extracted features for each user. For this proposal, the average user rating for each movie genre is determined once different movie genres are collected from the database. A collection of attributes describing the user model is built by integrating a set of user profile information with the average of their rating records to distinct genres.

The dimensions of the characteristics are subsequently reduced using the bee colony algorithm. In the design depicted in Figure 1, the target user is a user to whom we aim to deliver counsel, presuming he is not completely aware of his interests. So, the vector of features of this user is first constructed in accordance with the procedure of creating the user model in order to obtain a vector of length F for this user. The distance between the target user's attributes and all database users is then computed using the Euclidean distance criteria, and the most comparable to the target user is found using this criterion. Finally, the suggestion will be based on the interests of those who are most similar to the target user. Following that, we will go into the specifics of each step in the suggested strategy. These models will be saved as a matrix with dimensions *F *×  *N*, where N indicates the number of network users and F indicates the number of attributes that define each user. The dimensions of the characteristics are subsequently reduced using the bee cloning process.

In the design depicted in Figure 1, the target user is a user to whom we aim to deliver counsel, presuming he is not completely aware of his interests. For this purpose, the user's vector of features is constructed using the user model creation method, yielding a vector of length F for this user. The Euclidean distance criteria are then used to analyze the distance between the target user's qualities and those of all database users, and the most comparable to the target user is found.

The notations used in this study are listed in Table 2.

### 3.1. Preprocessing User Profile Data

Considering a network with a number of users, each user in the network has a collection of attributes known as a user profile. In the suggested technique, we will extract the personal information listed in Table 3 as the individual characteristics of each user from the profile and utilize it as one of the inputs to the proposed algorithm.

(Table 3 shows how the proposed approach uses three criteria to determine the characteristics of network users. Only the age feature has a numerical value, whereas the other individual features are described as nominal. Since similarity/distance algorithms cannot process non-numeric values, all non-numeric data are transformed to numeric during the preprocessing step. In the preprocessing step, how to change any of these characteristics is explained as follows:

*Gender*. Each person's gender characteristic can have one of three values: “male,” “female,” or “no value.” If the user has not provided the gender, the value is assumed to be zero. Otherwise, the numbers 1 and 2 replace the “man” and “female” values, respectively.

*Occupation*. In order to convert occupation attributes to numeric values, a unique list of all user occupations in the database is first created. It is assumed that this unique vector is *L*={*l*_1_,… *l*_*N*_}. We then assign to each of the unique occupations in the database a number corresponding to its location on the *L* vector. By doing this, the occupation ID *l*_1_ will be equal to one and the occupation ID *l*_*N*_ will be equal to *N*. Finally, we replace each user's occupation with the ID generated for that. It should be noted that if the user has not entered a value for the occupation attribute, we will replace it with a value of zero.

After converting all nominal qualities to numerical, the proposed strategy employs two independent methods to determine the difference between the nominal and numerical features of people. The age attribute is a numeric attribute for each individual in the database that is recorded as a number by year. This characteristic has a higher number of variations than the others. For example, we suppose that the average age of participants in a network is between 10 and 60. In this scenario, the maximum age gap will be 50 years; if this distance is equal to one for gender characteristics, the maximum age gap will be 50 years (zero distance for homosexuals and one for two people of the opposite sex). Because of these varied traits, calculating user similarity based on their attributes is challenging. The suggested solution employs the normalization of age data to tackle this difficulty. The following equation will be used to standardize the age attribute of every user in the network:

(1) $$\begin{array}{c} \overset{⟶}{N_{age}}=\frac{\overset{⟶}{age}-age_{\min}}{age_{\max}-age_{\min}}\text{.} \end{array}$$

In the above relation, ($\overset{⟶}{Age}$) is the age vector of individuals, age_min is the lowest age in the database, and age_max is the largest value in the database. The above relation causes the age values of individuals as a vector such as ($\overset{⟶}{N_{age}}$) in the range [0, 1]. This relationship causes age_min_ to be mapped to zero and age_max to be mapped to one. As a result of the preceding relationship, the greatest disparity between people's ages is one. However, in both cases, a different approach is used to determine the difference between two characteristics: gender and occupation. If two people have the same relevant attributes, we consider the distance to be zero; otherwise, we consider the distance to be one. This operation is defined by the following relationship:

(2) $$\begin{array}{c} d=\left\{\begin{array}{c} \begin{array}{cc} 0 & if\;f_{x}=f_{y} \end{array}\\ \begin{array}{cc} 1 & if\;f_{x}\neq f_{y}. \end{array} \end{array}\right. \end{array}$$

In the above relation, *f* identifies one of the nominal characteristics of gender or occupation. *f*_*x*_ represents the value of this feature for user *x* and *f*_*y*_ represents the value of this feature for user *y*. In this relation, *d* represents the distance between the two users *x* and *y* in terms of the feature *f*, and if the feature *f* of the two users is the same, this distance will be equal to zero; otherwise, it will be equal to one. Following this procedure for each user, the attributes of each individual will be described as a numerical vector of length 3. The proposed method's next step is to extract the features and utilize them to create the user model. This procedure will be described in the next section.

### 3.2. Extracting Features and Building User Models

Preprocessing user profile information yields a numeric matrix that describes the network user profile. Each row of this matrix describes a user's attribute, while each column indicates an attribute. As a result, if the number of network users is equal to *n*, the user attribute matrix will have dimensions *n* 3. It should be noted that content suggestions cannot be provided only on the basis of user attributes. Two users may, for example, have identical personal attributes (age, gender, etc.) yet differ in their hobbies. As a consequence, the recommended approach considers users' film scoring records as an extra piece of information when creating appropriate recommendations. The user model is then built by combining user profile information (personal characteristics) with his scoring records in various film styles (user interests). In order to extract the elements related to people's interests, a unique list of all cinematic genres in the database is originally produced. For example, doing so for the MovieLens database resulted in the extraction of 18 movie genres, which are listed below. Following the extraction of the database's list of cinematic genres, the users' scoring records for movies in each genre are retrieved, and the user's average rating for each genre is determined.

  “-Action”
  “-Adventure”
  “-Animation”
  “-Children”
  “-Comedy”
  “-Crime”
  “-Documentary”
  “-Drama”
  “-Fantasy”
  “-Film-Noir”
  “-Horror”
  “-Musical”
  “-Mystery”
  “-Romance”
  “-Sci-Fi”
  “-Thriller”
  “-War”
  “-Western”

**Figure 2:**
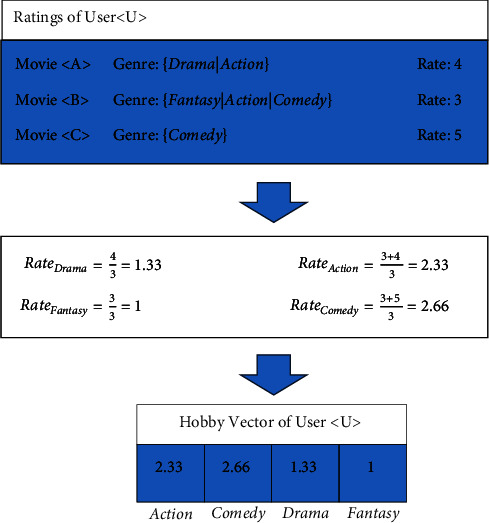
An example of the process of extracting the user interest vector in the proposed method.

Because a film may belong to more than one cinematic genre, the recorded rating for that film in all genres will be computed. Figure 1 depicts an example of the recommended method's user interest vector extraction approach (2).  Figure 2 illustrates all of the ratings obtained by the hypothetical user U. This user has three points, whereas the number of cinematic genres in the database is assessed to be four. In Figure 2, we can see that user U has rated for two movies in the comedy category, which we can use to get the average rating for this genre. We add these two points together and divide by the total number of user rating records. User U's average comedy genre rating will be 2.66 as a result of this. The average rating reported by users for each cinematic genre is determined using the procedure indicated in Figure 2, and a vector reflecting each user's interests in different genres is produced as a result.

Because there are 18 cinematic genres in the database, the length of the bar reflecting each user's choices will also be 18. By combining these attributes with the users' individual characteristics, the vector of each user's characteristics will be generated. This vector, which has 21 attributes, describes the collection of distinct qualities and interests of each person's (3) individual features and 18 average features of cinematic genres. Because the number of features returned for certain databases might be rather large, the bee colony optimization algorithm will be utilized in the following to choose the optimal characteristics in the user model. This method detects and eliminates unrelated aspects in the user model. This step can improve the accuracy and speed of the proposed method. The approach for selecting features using the bee colony optimization method will be discussed in the next section. The length of each response vector in the bee colony optimization technique is equal to the total number of features in the user model (N). As a result, each existing attribute is allocated a position in the optimization algorithm's solution vector. Each location can have a value of 0 or 1. If a location has a value of 0, that attribute is not selected in the current response; otherwise, the attribute corresponding to the current location is considered as the selected attribute. In order to fit each answer in the proposed algorithm by the bee colony optimization algorithm, first, the list of selected features is stored in sets such as *C*={*c*_1_,…., *c*_*i*_}. The present selection's fit is then determined, and the selection's optimality is assessed. In an optimization algorithm, the fit function is used to determine the degree of optimization of the response. We want to identify traits that best distinguish comparable user interest groups in the suggested strategy. One of the appropriate factors for selecting a feature is the use of the correlation parameter. This technique may be a suitable choice for rating the extracted attributes. Because, on the one hand, the relationship between each characteristic and user group is similar, and on the other hand, the correlation between characteristics is taken into consideration. Given a set of extracted qualities *x* and a set of categories *c*, the Pearson correlation relation [23] may be used to determine the correlation of the extracted features:

(3) $$\begin{array}{c} M_{x}=k\frac{\bar{r_{cf}}}{\sqrt{x+\left(x-1\right)\bar{r_{ff}}}}\text{.} \end{array}$$

In the above relation, *M*_*x*_ is the relation of the selected attribute set and $\bar{r_{cf}}$ is the mean of the linear correlation between the attribute set *x* and the categories *c*. It should be noted that in problems where the data classification is not clear (such as the current problem), $\bar{r_{cf}}=1$ is considered. Also, in the above relation, $\bar{r_{ff}}$ refers to the average of the linear correlation between the selected features. Therefore, the fitting function is the selection of features with maximum correlation between each other and the possible target categories. This goal is equivalent to maximizing (3), or in other words, minimizing the following equation:

(4) $$\begin{array}{c} f_{2,x}=\frac{1}{1+M_{x}}\text{.} \end{array}$$

In the proposed method, using a bee colony optimization algorithm, we select a subset of data that can minimize (4). This process is done through the following steps:

**Step 1 .**

(determining the problem limits). Determining the limits for each optimization variable is described in this section. Thus, each response vector is an integer vector, each of which is a member of the set {0, 1}.

**Step 2 .**

(preparation phase). In this step, all the vectors of the food source population (possible answers) are initialized by the scout bees as vectors${\overset{⟶}{x}}_{m}$,*m*=1, 2,…, *SN*; which in this vector, SN is equal to the size of the bee population. Since each member ${\overset{⟶}{x}}_{m}$is itself a vector containing the variable F, the goal is to find values in the vector ${\overset{⟶}{x}}_{m}$that produce the least value of the fitting function (equation (4)). The initialization of the vector ${\overset{⟶}{x}}_{m}$is done through the following relation [24]:

(5) $$\begin{array}{c} x_{m,i}=x_{\min}+R\times \left(x_{\max}-x_{\min}\right)\text{,} \end{array}$$

where *R* is a random member in the set {0, 1}.

**Step 3 .**

(search phase of worker bees). In this stage, the search bees look for new solutions with a better fit in the neighborhood of the vector${\overset{⟶}{x}}_{m}$. These bees evaluate the values in the vicinity of the vector ${\overset{⟶}{x}}_{m}$by Equation (9). In other words, the employed bees changed the list of selected features on the vector ${\overset{⟶}{x}}_{m}$to produce the vector${\overset{⟶}{v}}_{m}$. Then, they calculate the fit of the vector ${\overset{⟶}{v}}_{m}$ and check whether the features determined in ${\overset{⟶}{v}}_{m}$are more suitable than ${\overset{⟶}{x}}_{m}$ or not? If the answer is yes, the vector ${\overset{⟶}{x}}_{m}$will be removed and replaced by${\overset{⟶}{v}}_{m}$. The vector ${\overset{⟶}{v}}_{m}$is determined using the following equation [24]:

(6) $$\begin{array}{c} {\overset{⟶}{v}}_{m}=x_{mi}+\varphi_{mi}\left(x_{mi}-x_{ki}\right)\text{.} \end{array}$$

In the above relation, *x*_*k*_is a random vector selected from the population, *i* is a random location selected on the response vectors, and *φ*_*mi*_is a random value in the interval [0, 1].

**Step 4 .**

(onlooker bees evaluation phase). Employed bees provide the control bees with information about the responses found. Observer bees calculate the probability of selecting each answer and select the appropriate answer to search for in the next steps. The probability of the vector ${\overset{⟶}{x}}_{m}$ is calculated by the observing bee using the following equation [24]:

(7) $$\begin{array}{c} P_{m}=\frac{fitness\left({\overset{⟶}{x}}_{m}\right)}{\sum_{i=1}^{SN}fitness\left({\overset{⟶}{x}}_{i}\right)}\text{.} \end{array}$$

As previously stated, the solutions are chosen based on the probability value of equation (7). The answers are determined using the Roulette wheel algorithm. This technique selects the most likely solutions for use in the next population, but a small number of nonoptimal replies are also pushed to the next cycle to preserve the problem's comprehensiveness.

**Step 5 .**

(watch bee search phase). In this step, responses that remain unchanged after a certain number of cycles are randomly reset by watch bees.

**Step 6 .**

(evaluation of results). If the number of search cycles reaches the specified maximum value, the algorithm terminates. Otherwise, the search algorithm for the new cycle is repeated from step 2.

In the final step of the proposed method, the selected features will be used to provide a recommendation. In the following sections, we will discuss the recommendation process based on the extracted features for users.

### 3.3. Providing Recommendations Based on the Extracted Characteristics

The last step in the proposed method is to provide a recommendation based on the features selected in the previous step. If we consider the number of network users to be N, the whole database will be described as a matrix with dimensions *N*  × *F*. Each column of this matrix represents a feature, while each row indicates a user's unique features and interests. To provide counsel to a user like *u*, the user attribute vector *u* is first generated, and then the user Euclidean distance *u* is computed from all database users. It should be mentioned that equation (2) is utilized to determine the distance between users' gender and occupation attributes. The following equation is used to compute the distance between user *u* and *v*:

(8) $$\begin{array}{c} Dist\left(u,v\right)=\sqrt{\overset{F}{\underset{i=1}{\sum }}{\left(u_{i}-v_{i}\right)}^{2}}\text{.} \end{array}$$

In the above equation, F represents the number of database features and *u*_*i*_ represents the value registered for the user's *i*th feature. After determining the distance between user *u* and other users, the suggestion will be based on the database users that are closest to *u*. As a consequence, after identifying the user with the shortest distance, his database video scoring records will be analyzed, and the *R* movie with the highest recorded rating will be recommended to user *u*. R is the number of suggestions parameter, which is determined by the system in this case.

## 4. Implementation and Results

In this section, the proposed method is implemented using MATLAB software and the performance of the proposed method is examined. The efficiency of the proposed method is also compared with other learning models. MovieLens database was used in the experiments. MovieLens is a web and virtual community-based service that recommends movies to its users. The system contains about 11 million rankings for about 8,500 films and was created in 1997 by GroupLens Research, a research laboratory in the Department of Computer Science and Engineering at the University of Minnesota, to collect research data on personal recommendations. In this research, Movielens 1M version of this database has been used to evaluate the proposed method.  Table 4 lists a summary of the specifications of this database [25].

This database describes each user's profile through 10 attributes. In the proposed method, only three features: age, gender, and occupation are used and other features mentioned in the user profile are ignored. Also, for each movie in the database, the features of the ID, name, year of release, and genre are available. User ratings for movies in the MovieLens 1M database do not have a timestamp. As a result, the model cannot be built and recommended in two separate time periods. As a result, double cross-evaluation is used to assess the suggested algorithm's performance. The database is partitioned into ten subgroups in this assessment technique, and the modeling and recommendation procedure is performed ten times. In each experiment, 9 subsets of the database are used to create the model and the user similarity matrix, with the remaining subset being utilized to assess the proposed method's performance in proposing to users. In other words, all of the points recorded for the films by each user are separated into 10 groups. The user model and their similarity matrix are constructed using 90% of the ratings (9 parts) each time the experiment is run, and the suggestions supplied by the proposed technique are extracted based on this similarity matrix. The suggested algorithm's output is then compared to the remaining 10% of real ratings. In each comparison, one of the following scenarios might occur:

- The proposed algorithm proposes a movie that is available in the remaining 10% of the user-recorded ratings (in other words, a rating higher than 3 for the user-suggested movie is recorded). In this case, the recommended film is a true positive output and is added to the TP set.
- The proposed algorithm has suggested a movie that is not available in the remaining 10% of the points recorded by the user. In this case, the recommended connection is a false positive output and is added to the FP set.
- A set of videos is available in the remaining 10% of user-rated ratings that are not recommended by the proposed algorithm (in other words, the suggested algorithm cannot recommend user-favorite movies with a rating higher than 3). All of these ignored videos are added to the FN collection as false negatives.

Using the above results, precision and recall criteria can be used to evaluate the performance of the recommender system. The precision criterion determines the ratio of the correct outputs of the recommender algorithm, which is calculated as follows:

(9) $$\begin{array}{c} precision=\frac{TP}{TP+FP}\text{.} \end{array}$$

The recall criterion also represents the proportion of the user's favorite movies that are correctly recommended by the proposed algorithm. This criterion is calculated using the following equation:

(10) $$\begin{array}{c} recall=\frac{TP}{TP+FN}\text{.} \end{array}$$

In evaluating the proposed method, three different scenarios have been considered for conducting the experiments, which will be discussed later:

1. *Changing the Number of Recommendations*. In this experiment, the number of movies recommended to the tested users is changed in the range of 1 to 5 and the efficiency of the proposed method for these changes will be studied.
2. *Changing the Number of Users*. In this scenario, we will change the number of users used to perform the recommendation in the range of 2000 to 6000 users. In each iteration, the list of users used to evaluate the performance of the proposed method will be the same as the compared methods.
3. *Changing the Number of Recommendable Items (Videos)*. The purpose of this experiment is to evaluate the performance of the proposed method in terms of changes in the number of items recommended to users. For this purpose, the number of database videos is changed in the range of 1000 to 3883 and the precision and recall criteria for these changes will be examined.

### 4.1. Performance of the Proposed Method Considering the Number of Recommendations

**Figure 3:**
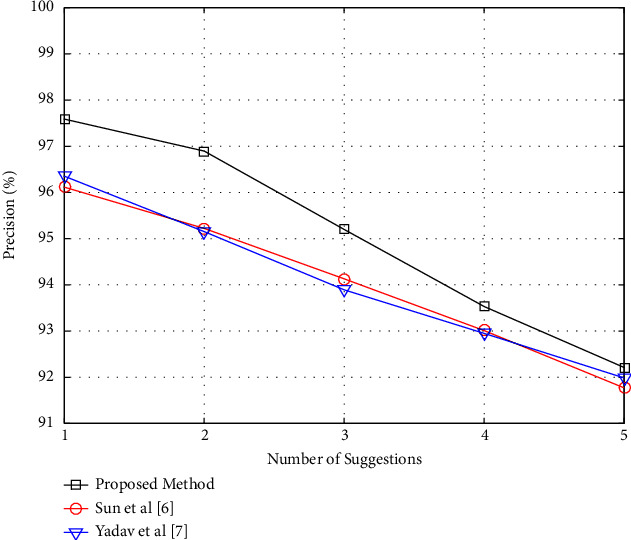
Precision per number of recommendations in the proposed method.

In this experiment, the number of items (videos) recommended by the proposed algorithm is modified, and the precision and recall requirements for each case are calculated. The amount of recommendations is the number of videos that the proposed approach recommends each user view based on their similarity matrix. The suggested number might range between one and five. It's worth mentioning that all database users and videos were utilized to offer suggestions in this experiment.  Figure 3 displays a graph of variations in the precision criterion as a function of the number of recommendations in the proposed approach.

**Figure 4:**
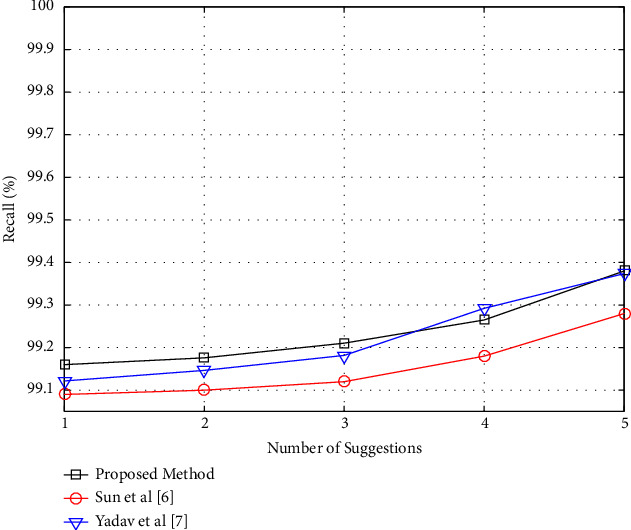
Recall per number of recommendations in the proposed method.

The outcomes of the proposed method are compared to the procedures provided in references [6, 7] in both of these graphs. These data imply that the recommended technique generates superior results. The precision chart in Figure 3, on the other hand, shows a downward trend, and the recall chart in Figure 4 indicates an upward trend. This feature suggests that a range of recommendations may be effective in increasing precision. The fact that the proposed technique decreases precision while increasing the number of recommendations indicates that it frequently prioritizes appropriate offers. A good recommender algorithm should have this attribute. As seen in Figure 3, the proposed technique prioritizes more acceptable films while giving less weight to alternative choices (in other words, as the number of recommendations grows, they are more likely to be included). The recall criteria, on the other hand, increase as the number of suggestions of the suggested technique in Figure 4 grows. This characteristic demonstrates that as the number of suggestions grows, a greater percentage of the user's favorite videos is successfully retrieved. Also, Figure 4 shows a graph of changes in the recall criteria for this experiment.

### 4.2. Performance of the Proposed Method considering the Number of Users

**Figure 5:**
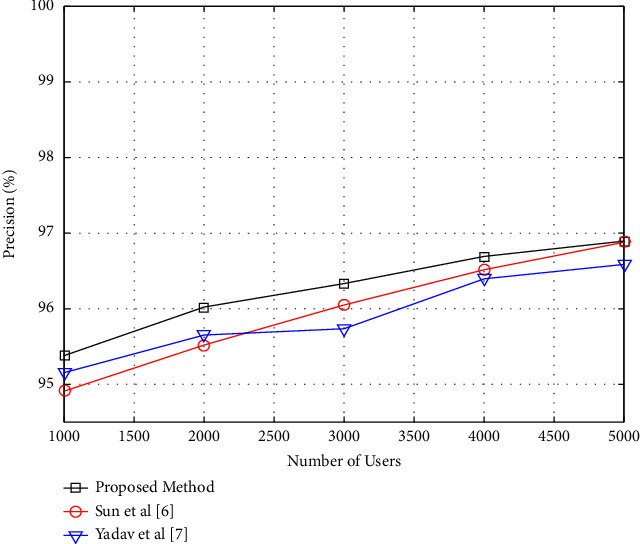
Precision versus the number of users.

**Figure 6:**
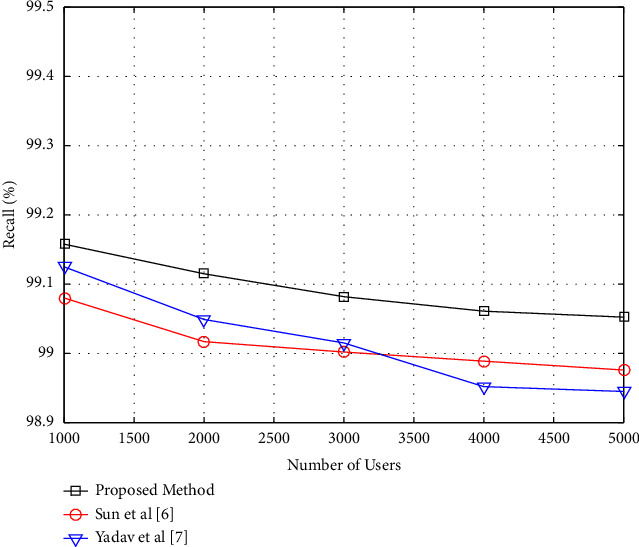
Recall versus the number of users.

This section examines how the number of users impacts the performance of the recommended technique. The purpose of this experiment is to investigate how the proposed approach reacts to changes in the problem's dimensions. In this case, the proposed algorithm generates two suggestions. In addition, all of the movies in the database were used as suggested items. The number of database users is then modified in the range of 2000 to 6000, while the influence of dimensional changes on the performance of the suggested approach is tested by continually evaluating these parameters. The graphs showing changes in precision and recall criteria for changes in the number of users in the proposed technique are illustrated in Figures 5 and 6, respectively.

As seen in Figures 5 and 6, the precision criteria rise while the recall criterion falls (6). As the number of users increases, the recommending system will get more information on the link between users' traits and their favorite movies. As a consequence of this data, the proposing system will have a better understanding of the problem and will be able to give more likely recommendations. However, as the number of users and the network expand, a greater proportion of users' rights and interests will be destroyed during the testing phase, and the recommending system will be forced to uncover more hidden interests through recommendation. In other words, as the network expands in size, so does the number of test samples. The number of recommendations, on the other hand, is limited to two. As a result, it is natural that in this situation, the criterion of recall is decreasing. However, in this experiment, the proposed method has a better performance than the compared methods.

### 4.3. Performance of the Proposed Method considering the Number of Movies

**Figure 7:**
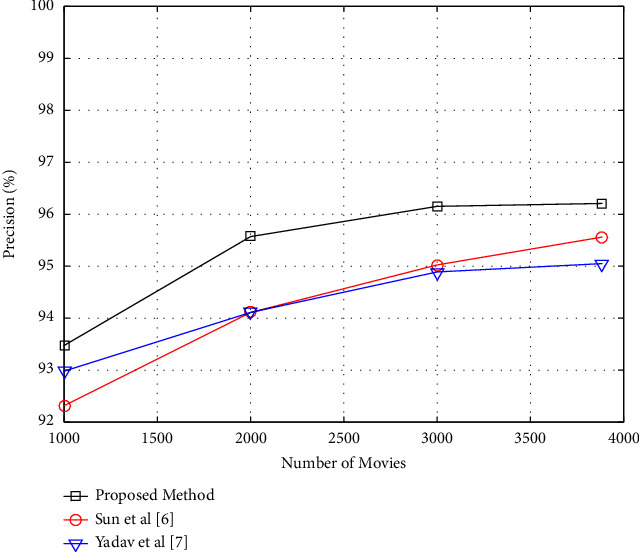
Precision of recommendation versus the number of recommendable movies.

**Figure 8:**
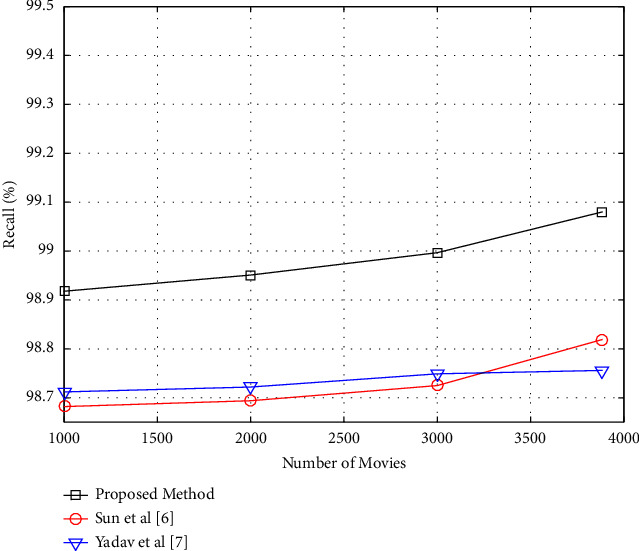
Recall values versus the number of recommendable movies.

The goal of this experiment is to see how well the suggested strategy performs as the number of things recommended to users fluctuates. The amount of database videos will be modified from 1000 to 3883 for this purpose, and the precision and recall criteria for these modifications will be investigated. The number of suggestions produced by the suggested algorithm is assumed to be two in this instance. In addition, all database users were utilized to create the model and test the recommender system. In the suggested technique, Figure 7 depicts a graph of changes in the precision criteria as a function of the number of movies. The precision values will increase as the number of database films grows, as illustrated in Figure 7. Because as the number of films in the database grows, so does the variety of options for offering a suggestion, and as a result, the likelihood of providing an acceptable recommendation to the user grows as well. The suggested technique may be more successful in delivering suggestions than the comparison method, based on the findings of this experiment. In the suggested technique, Figure 8 depicts a graph of changes in the recall criterion as a function of the number of films.

As the number of database movies grows, so will the recall values, as seen in Figure 8. Because the suggested strategy would generate a more complete model of users' interests in different genres by increasing the quantity of videos in the database. As a consequence, the suggested technique has a better likelihood of correctly extracting users' favorite movies.

Table 5 summarizes the average precision and recall values of the proposed method with compared recommender systems during experiments. In this table, the computational costs of the methods, are also compared. These experiments have been executed on a personal computer, running ×64 version Microsoft Windows 10 on an Intel core i7 processor with the processing power of 3.2 GHz and 16 gigabytes of RAM.

In Table 5, the model construction time refers to the average time needed for constructing a user model and is calculated by dividing total model construction time of recommender system by the number of users. The recommendation time also refers to the average computational time required for providing a recommendation through the constructed model.

According to the mean values in Table 5, the proposed strategy outperforms the compared methods, boosting precision by 1.39% and recall by 0.8%. These results indicate that the proposed model is a cost-effective method, which can be deployed in real-world scenarios. According to these results, the proposed scheme can reduce the model construction time and recommendation time by 57.83% and 54.71%, respectively. This improvement can be attributed to using reduced features that are provided by the ABC optimization algorithm.

## 5. Conclusion

This research proposed a revolutionary algorithm for recommending content to consumers in video provider networks. The three key processes in the proposed system for providing customers with advice are “preprocessing user profile information,” “building profile structure and extracting features,” and “recommending based on the similarity of users' models.” By combining a set of user profile qualities with the average of their rating records to separate genres, the proposed approach [25] generates a collection of attributes that describe the user model. According to the experiments, the computational cost of the proposed method is very low, which makes it a more efficient way to be used in huge networks compared to the previous solutions. In addition, using the ABC algorithm for selecting optimal features is effective in reducing the processing time and improving the precision of the recommender.

One of the limitations of the proposal is that the parameters of the number of recommendations and the number of features (in the ABC algorithm) must be determined empirically by the user. In future works, the task of determining suitable values for these parameters can be assigned to the optimization algorithm. Another aspect for improving the efficiency of the proposed method is clustering users, which may lead to a better approach for selecting the most similar users with lower computational complexity. Thus, it can be studied in future works.

## Figures and Tables

**Table 1:** Summary of the related studies.

Author(s)	Year	Research objective	Research method
Sun et al. [6]	2017	User-centric content recommendation to users of social network	Clustering based on the common interests and communication analysis
Yadav et al. [7]	2021	Movie recommendation	PCA (for feature reduction) and K-means clustering (for finding similar users)
Arojo et al. [8]	2019	Modelling the behavior of social media users based on dynamic recommender structure	Two successive classifiers for detecting similar users and their favorite contents
Doonan et al. [9]	2019	Clustering-based dynamic recommender structure	A new similarity criterion for clustering users
Dakotor [10]	2015	Presenting a new similarity criterion for selecting similar users with the target	Calculating similarity based on frequency and duration of user presence on a content
Gorji et al. [11]	2020	Hybrid recommender structure for websites	Application-based and content-based analysis of user data
Gunawardana and Shani [12]	2015	Hybrid recommender structure for websites	Distributed learning automata (for structure analysis) and Markov model (for recommendation)
Kung et al. [13]	2019	Hybrid recommender system for social networks	Modelling users motion among contents and behavior analysis
Latahabai et al. [14]	2017	Application-based dynamic recommender for social networks	Automated rules and indexing to create automated profiles for users
Parish et al. [15]	2018	Content-based recommender for social networks	Analyzing contents and generating profiles based on user behavior
Toth and Lengyel [16]	2019	Improving personalized results of search engines	Generating and continuous updating user profiles based on behavioral history

**Table 2:** Notations used in this study.

Symbol	Description
*φ*_*mi*_	Random value in the interval [0, 1] for modifying a solution in ABC algorithm
Dist(*u*, *v*)	The Euclidean distance between users *u* and *v*
*F*	The number of features in the database
$fitness\left({\overset{⟶}{x}}_{m}\right)$	The calculated fitness value for solution vector ${\overset{⟶}{x}}_{m}$ in ABC algorithm
*f*_min_	The minimum value of feature *f* in database
*f*_max_	The maximum value of feature *f* in database
FN	The number of false negative samples
FP	The number of false positive samples
*M*_*x*_	Pearson correlation coefficient of feature *x*
N	The number of network users
$\overset{⟶}{N_{f}}$	Normalized vector of feature *f*
*P*_*m*_	The probability of selecting a solution ${\overset{⟶}{x}}_{m}$ in the evaluation phase of ABC algorithm
$\bar{r_{cf}}$	Average of the linear correlation between the attribute set *f* and the categories *c*
$\bar{r_{ff}}$	Average of the linear correlation between the features of set *f*
SN	The population size in the ABC algorithm
TP	The number of true positive samples

**Table 3:** A set of personal characteristics used to describe the user profile in the proposed method.

Row	Title	Feature type
1	Age	Numerical
2	Sex	Nominal
3	Occupation	Nominal

**Table 4:** MovieLens 1M database specifications [25].

Property	Value
Year of publication	2012
Number of users	6040
Number of movies	3883
Number of points registered by users	1000209

**Table 5:** Comparison of the average precision and recall of the proposed method in all experiments.

	Average precision	Average recall	Average model construction time (s)	Average recommendation time (s)
Suggested method	96,159	99,438	0.317	0.0015
Sun et al. [6]	94,760	98,263	0.858	0.0017
Yadav et al. [7]	94,777	99,010	0.669	0.0650
Average improvement (%)	+1.39%	+0.80%	−57.83%	−54.71%

## Data Availability

Data are available and can be provided upon request to the corresponding author (shafieian@iribu.ac.ir).
